# Standardizing Visual Control Devices for Tsetse Flies: East African Species *Glossina fuscipes fuscipes* and *Glossina tachinoides*

**DOI:** 10.1371/journal.pntd.0003334

**Published:** 2014-11-20

**Authors:** Francis Oloo, Andrea Sciarretta, Mohamed M. Mohamed-Ahmed, Thomas Kröber, Andrew McMullin, Steve Mihok, Patrick M. Guerin

**Affiliations:** 1 Tsecon Consultants, Nairobi, Kenya; 2 Department of Agriculture, Environmental and Food Sciences, University of Molise, Campobasso, Italy; 3 College of Veterinary Medicine, Sudan University of Science and Technology, Khartoum-North, Sudan; 4 Institute of Biology, University of Neuchâtel, Neuchâtel, Switzerland; 5 Independent Scientist, Russell, Ontario, Canada; IRD/CIRDES, Burkina Faso

## Abstract

**Background:**

Riverine species of tsetse are responsible for most human African trypanosomiasis (HAT) transmission and are also important vectors of animal trypanosomiasis. This study concerns the development of visual control devices for two such species, *Glossina fuscipes fuscipes* and *Glossina tachinoides*, at the eastern limits of their continental range. The goal was to determine the most long-lasting, practical and cost-effective visually attractive device that induces the strongest landing responses in these species for use as insecticide-impregnated tools in vector population suppression.

**Methods and Findings:**

Field trials were conducted in different seasons on *G. f. fuscipes* in Kenya, Ethiopia and the Sudan and on *G. tachinoides* in Ethiopia to measure the performance of traps and 2D targets of different sizes and colours, with and without chemical baits, at different population densities and under different environmental conditions. Adhesive film was used to enumerate flies at these remote locations to compare trapping efficiencies. The findings show that targets made from black and blue fabrics (either phthalogen or turquoise) covered with adhesive film render them equal to or more efficient than traps at capturing *G. f. fuscipes* and *G. tachinoides*. Biconical trap efficiency varied between 25% and 33% for the two species. Smaller 0.25 m×0.25 m phthalogen blue-black targets proved more efficient than the regular 1 m^2^ target for both species, by over six times for *Glossina f. fuscipes* and two times for *G. tachinoides* based on catches per m^2^. Overall, targets with a higher edge/surface area ratio were more efficient at capturing flies.

**Conclusions/Significance:**

Taking into account practical considerations and fly preferences for edges and colours, we propose a 0.5×0.75 m blue-black target as a simple cost-effective device for management of *G. f. fuscipes* and *G. tachinoides*, impregnated with insecticide for control and covered with adhesive film for population sampling.

## Author Summary

The riverine tsetse *Glossina fuscipes fuscipes* and *Glossina tachinoides* are among the principal vectors of African trypanosomiasis. Their range stretches from West across Central to East Africa, with isolated populations in Sudan and Ethiopia. Population suppression is one of the most effective methods to control disease transmission and has led to the development of visually attractive insecticide-impregnated traps and targets for *palpalis* tsetse species for over half a century. We describe field experiments made in different seasons in Sudan, Ethiopia and Kenya to establish the most efficient and long-lasting object that induces the strongest landing responses in *G. f. fuscipes* and *G. tachinoides*. Independent of season and country, targets made from black and blue fabrics (either phthalogen or turquoise) covered with adhesive film render them equal to or more efficient than traps at capturing *G. f. fuscipes* and *G. tachinoides*. Biconical trap efficiency varied between 25% and 33% for both species. As landings per unit area on smaller phthalogen blue-black targets were significantly higher than landings on corresponding 1 m^2^ targets, we propose a 0.5 m high×0.75 m wide blue-black target as a practical cost-effective device for management of *G. f. fuscipes* and *G. tachinoides* populations.

## Introduction

**Figure 1:**
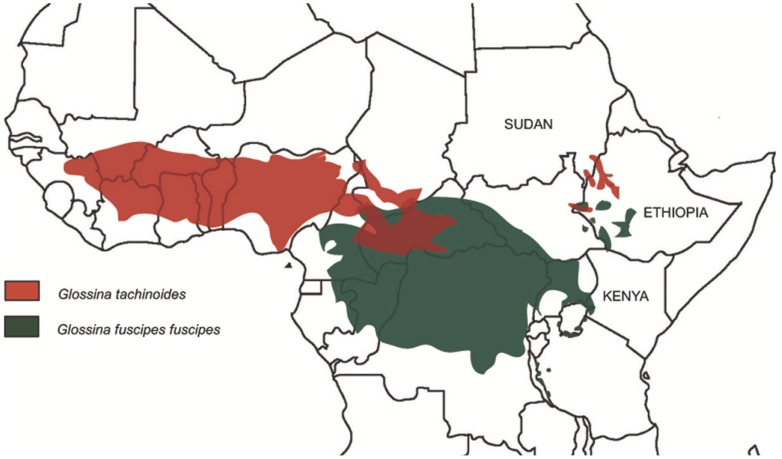
Main distribution of *G. tachinoides* and *G. fuscipes fuscipes* based on data in Rogers and Robinson (2004) [**2**].

Among tsetse flies (*Diptera*, *Glossinidae*) the *palpalis* group is responsible for most human African trypanosomiasis (HAT) transmission; with 90% of new sleeping sickness cases being transmitted by species from this group [1]. This study concerns the development of visual control devices for two of these species, *Glossina fuscipes fuscipes* (*G. f. fuscipes* Newstead 1910) and *Glossina tachinoides* (*G. tachinoides* Westwood 1850). *G. f. fuscipes* is found in Central Africa in the Cameroon, Gabon, the Republic of the Congo, the Democratic Republic of the Congo (DRC), the Central African Republic, Chad, Sudan and South Sudan, extending east to the northern shores of Lake Victoria in Uganda and western Kenya. Isolated populations also occur in Tanzania, Sudan and Ethiopia. *G. tachinoides* is distributed across West Africa in a zone stretching from Guinea eastwards through northern Nigeria, Niger and southern Chad to the Central African Republic. Isolated populations also occur in the Sudan, South Sudan and Ethiopia (Figure 1 [2]).

Both species are found naturally in riverine and lacustrine habitats. They are vectors of the acute form of HAT caused by *Trypanosoma brucei rhodesiense (Kinetoplastida, Trypanosomatidae)* and of the chronic form caused by *T. b. gambiense*. They are also vectors of African animal trypanosomiasis (AAT), which causes three million cattle deaths per year in Africa [3]. The costs of AAT alone to African economies is tremendous; a recent study estimates that the total maximum benefit of eliminating the disease in six East African countries amounts to nearly US$ 2.5 billion [4].

After an alarming rise in cases of HAT in the 1990s (40,000 cases reported and ∼300,000 undiagnosed and untreated in 1998 [5], increased treatment and coordinated vector control has led to a steady decline. In 2009, the number of reported new cases dropped below 10,000 for the first time in decades. Actual new cases are estimated at about 30,000 per year (7,197 reported new cases in 2012 [5]. Despite these successes, sleeping sickness remains a major public health problem in large parts of sub-Saharan Africa. The most recalcitrant foci of HAT occur in areas occupied by *G. f. fuscipes* and *G. tachinoides* [6]. Most new cases are reported from the Democratic Republic of Congo, the Central African Republic, South Sudan and Chad [5].

Riverine tsetse such as *G. f. fuscipes* and *G. tachinoides* have been able to adapt to peri-domestic habitats [7]. Hence, they are sometimes important vectors in degraded habitats modified by humans [8]. For example, *G. f. fuscipes* is the main vector of HAT in the heavily-populated Lake Victoria basin where *G. pallidipes* is also involved in transmitting the disease [9]. Previous control campaigns against both species in western Kenya included spraying waterside vegetation with DDT and block spraying dieldrin along transects in the savannah 100 m apart [10]. Likewise, *G. tachinoides* occurs in the same habitat with *G. palpalis gambiensis*, one of the main vectors of HAT in West Africa [11]. *G. tachinoides* is a feeding opportunist and switches readily to domestic animals and people in the absence of natural hosts [12]. One study in Nigeria found that 43% of blood meals had been taken from humans [13], and it has recently been shown to have a higher vectorial capacity than *G. p. gambiensis* [14].

One of the many control strategies widely used for riverine tsetse control is the large-scale deployment of visually attractive traps and targets impregnated with a long-lasting insecticide [15]. This is an affordable and efficient method of vector control. *G. f. fuscipes* is currently the focus of such an effort in Eastern Africa led by the Pan African Tsetse and Trypanosomiasis Eradication Campaign (PATTEC). This project is underway at the eastern limit of its continental range along the shores of Lake Victoria in an area with complex HAT epidemiology [16], where *G. f. fuscipes* is the main vector for HAT transmission and was responsible for the major historical outbreaks of 1900–1920 and 1976–1989 [17]. Considerable work has been done recently to improve visually attractive devices for *G. tachinoides* in West Africa [9], [18]–[22] and for *G. f. fuscipes* in Kenya [9], [18], [19], [23], but little work has been done for the isolated populations in East Africa.

Within the Africa-wide WHO-TDR initiative to develop standardised visual control devices for tsetse, we set out to evaluate practical devices for isolated populations of *G. f. fuscipes* and *G. tachinoides* in Sudan and Ethiopia (Figure 1). Our aim was to improve efficiency and cost-effectiveness in the context of existing control tools, usually traps, in these countries [24]. Field trials were also made for *G. f. fuscipes* in Kenya to compare findings with this species in the Sudan and Ethiopia. A second objective of these coordinated trials was to test whether behavioural responses were the same under diverse circumstances as in core regional populations studied here and elsewhere in Africa. The trials were based on trap/target/bait technology in actual use at each location following a coordinated experimental protocol throughout Africa targeting different tsetse vectors [22], [25], [26].

Trials were conducted in different seasons, in different environments and under a wide variety of tsetse population densities in the Sudan, Ethiopia and Kenya to measure the performance of pyramidal, monoconical and biconical traps and targets of different sizes in phthalogen blue cotton and selected alternative blue fabrics. A simple enumeration method (adhesive film) was used at these remote locations to compare trapping efficiencies of devices made of well-characterized, colour-fast fabrics. The relative performance of devices was also compared with and without a chemical bait. The goal was to determine the most practical and cost effective device/material that would induce the strongest landing response in *G. f. fuscipes* and *G. tachinoides* for future use throughout their range in large-scale population suppression of these tsetse spp. with insecticide-impregnated, visually-attractive devices.

## Materials and Methods

### Study sites

Studies on *G. f. fuscipes* were conducted over four years (2009–2012) at three sites located in three countries in Eastern Africa: Kenya, Ethiopia and the Sudan. Studies on *G. tachinoides* were undertaken at one site in Ethiopia in 2010 and 2012. A brief description of each site is given below.

#### Kenya (*G. f. fuscipes*)

**Figure 2:**
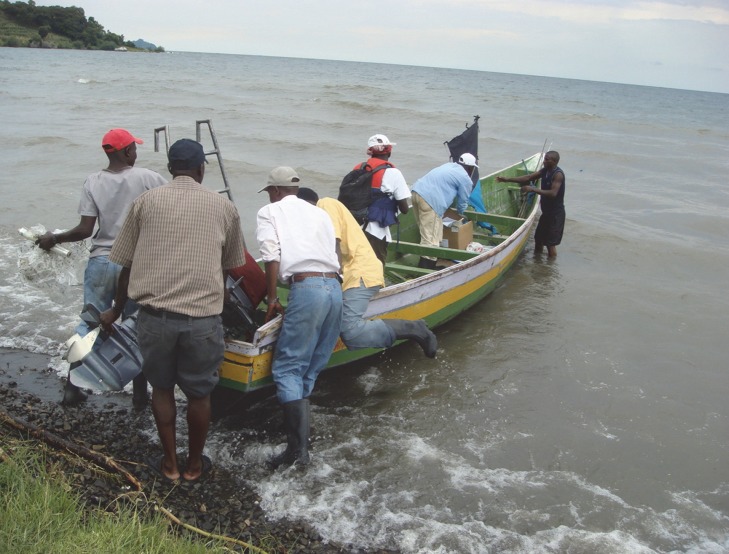
Placing adhesive film on bi-coloured target, Mfangano Island, Lake Victoria, Kenya.

The experimental site on Mfangano Island, Lake Victoria (S 0°45′27″ E 33°57′31″) was a mosaic of indigenous scrub and forest interspersed by plantations of maize, cassava, sweet potatoes and bananas. Experiments were conducted at the water's edge, along the shore in July 2009 (wet season), February 2010 (dry season), and January 2011 (dry season) (Figure 2).

#### Ethiopia (*G. f. fuscipes*)

A mosaic of fragmented gallery forest and thickets, bordered by cultivated fields, along the Gibe river, a tributary of the Omo river in south-west Ethiopia (N 8°15′43.65″ E 37°31′47.75″). The most abundant wild animals present were hippopotamus, with crocodiles and monitor lizards. Domestic animals, mainly cattle and goats, were also present but in small numbers. Studies were conducted in December 2010 (wet season) and February 2012 (dry season).

#### Ethiopia (*G. tachinoides*)

Intact gallery forest along the Didessa River in western Ethiopia (N 8°41′09.06″ E 36°25′00.27″). The river was bordered by tall elephant grass with cultivated fields nearby. Wild hosts in the area were mainly hippopotamus, crocodiles, buffaloes and gazelles. Herds of domestic cattle are regularly watered along the watercourse. Studies were conducted in November 2010 (wet season) and January 2012 (dry season).

#### Sudan (*G. f. fuscipes*)

A densely populated area still supporting fragmented gallery forest along the Khor Yabus River in south-eastern Sudan (N 9°57′06.32″, E 34°10′49″). Wild animal hosts were scarce but domestic animals (sheep, goats and donkeys) were abundant. Studies were conducted in March–April 2010 (dry season).

### Catching devices, materials and baits

Four catching devices were routinely tested: standard biconical [27] and pyramidal [28] traps, and two target designs: a regular 1 m^2^ square cloth target and a traditional Kenyan target of 1.5 m^2^ (1 m high by 1.5 m wide), both made of equal vertical rectangles of blue and black. Three blue fabrics were tested: C180 phthalogen blue 100% cotton, 180 g/m^2^, TDV, Laval, France (spectral reflectance peak at 460 nm as measured with a Datacolor Check Spectrophotometer, Datacolor AG, Dietlikon, Switzerland) and referred to here as the standard fabric; turquoise blue Q10067 65% polyester/35% viscose, 234 g/m^2^ Sunflag, Nairobi, Kenya (reflectance peak at 480 nm). A local blue fabric (origin unknown), 100% polyester, 175 g/m^2^ (reflectance peak at 420 nm) was also tested as a single kind of target in Ethiopia. One black fabric (Q15093 100% polyester, 225 g/m^2^, Sunflag, Nairobi) was used for all devices. In Ethiopia in 2012, a monoconical trap [29], and several smaller target sizes (divided vertically into equal blue and black sections) were also tested for *G. tachinoides* (0.25 m^2^ square and a 0.375 m^2^ horizontal oblong targets) and for *G. f. fuscipes* (0.25 m^2^ and 0.0625 m^2^ square targets).

**Figure 3:**
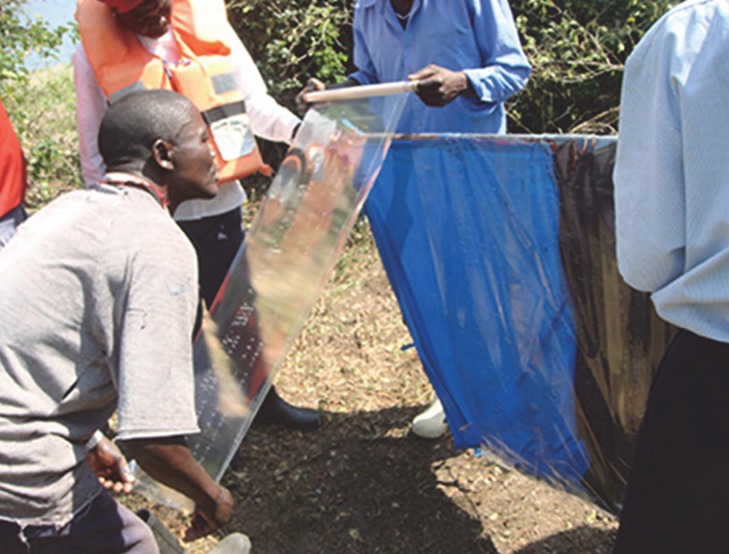
Transporting devices between trapping positions around Mfangano Island, Lake Victoria, Kenya.

To monitor the numbers of tsetse landing on targets, one sided adhesive film (Rentokil FE217, UK) was attached to both sides of the targets (Figure 3). There is an additive in the glue to protect it from ultra-violet light and spectra reflectance measurements show nearly all wavelengths in the UV range are absorbed. Reflectance of other wavelengths remains unchanged (Figures S1, S2, S3). The film was also attached to the cloth component of traps in some experiments to enumerate flies that land on traps but may not be captured in the cage. In one set of trials in Ethiopia, a 1×1 m square of adhesive film alone (without any cloth backing) was compared to cloth targets with adhesive film attached to both sides to ascertain whether adhesive film in itself attracts tsetse.

A 1∶4∶8 mixture of 3-n-propylphenol (P), 1-octen-3-ol (O), and p-cresol (C) was used as an attractant for experiments comparing baited devices based on efficacy for several tsetse (Ubichem Research LTD, Budapest/Hungary, global purity of 98%). Sachets of 4 cm×5 cm 500 gauge/0.125 mm polyethylene containing 3 g of the mixture were placed below the catching devices, 10 cm above ground, alongside a 250 ml bottle buried up to the shoulders containing acetone (A) with a 2 mm aperture in the stopper. This combination is termed POCA bait and was made up as per Torr *et al.*, (1997) [30].

### Experimental design

#### Best trapping device and blue material

To determine the best catching device and the most attractive blue fabric, experiments compared four to six devices at any given site in a Latin square design of days×sites×treatments, with three simultaneous replicates. Trap sites were always >100 m apart. Flies were counted after 24 hours at each position. The core devices tested were: biconical traps and pyramidal traps, and regular and traditional Kenyan targets in standard blue cotton or turquoise blue polyester/viscose.

#### Performance of POCA-baited trapping devices

The four to six device experiments were repeated using the POCA bait after the unbaited trial was completed in the same general area, with trapping positions >200 m apart because the attractants can be effective up to 100 m downwind. The experiments with POCA had the sole objective of trying to determine whether baiting changed the performance ranking of the devices/fabrics, as it was not logistically possible to undertake direct comparisons between unbaited and baited devices at the study sites.

#### Best landing device and optimal target size

To assess the efficiency of 3-d traps versus 2-d targets as landing devices, catches in biconical traps with adhesive film on the cloth component were compared to a series of different target sizes covered with adhesive film on both sides. In Kenya regular 1 m^2^ and1.5 m^2^ traditional Kenyan targets were evaluated, whereas in Ethiopia regular 1 m^2^ and 0.25 m^2^ square targets were compared to a biconical trap, along with a 0.375 m^2^ horizontal oblong target (*G. tachinoides* only) and a 0.0625 m^2^ square target (*G. f. fuscipes* only).

It was only possible to place the adhesive film on the outer blue surface (excluding the four fly entry holes) of the biconical trap. All catching devices in these experiments were made of standard phthalogen blue cotton. In this study we were interested in comparing the killing efficiency of the different devices (i.e. actual numbers landing) to evaluate them as control devices, so flies caught in the cage of the traps were not included in the total for this comparison as they had not landed on the device. Biconical traps not treated with the adhesive film were included as controls to estimate trap efficiency (percentage flies caught in control compared to those caught by the trap with adhesive film, i.e. on adhesive film and in the cage). The surface areas of adhesive film on the different devices are summarized in Table 1.

**Table 1:** Dimensions and surface areas of trapping devices.

Trap/target type	Shape	Surface area of adhesive film (m^2^)
Biconical trap	-	0.700
1.00 m×1.50 m Kenyan target	horizontal oblong	3.000
1.00 m×1.00 m regular target	square	2.000
0.50 m×0.50 m target	square	0.500
0.25 m×0.25 m target	square	0.125
0.50 m×0.75 m target	horizontal oblong	0.750

In Kenya, landing by *G. f. fuscipes* on the targets was recorded as a function of height, based on three equal horizontal strips each 33 cm high, (i.e. top third, middle third, bottom third). The aim was to determine whether there was a preferential landing height on blue or black, and to assess any difference between the landing responses of males and females. As the data suggested a preference by *G. f. fuscipes* for the borders of targets, landing distribution was tested against the length of the outer edges for each horizontal section. The outer perimeter lengths of the upper and lower blue and black sections were 0.83 m and 1.08 m for the 1 m^2^ and 1.5 m^2^ targets, respectively. The outer perimeter lengthfor the middle blue and black sections of both target sizes was 0.33 m.

#### Statistical analysis

In all trials randomization was set up using design.lsd in the package agricolae [31], R version 3.01 [32]. Data were analysed using a linear model including the following additional packages: MASS [33] and multcomp [34]. Analysis was performed on log (x+1) transformed data including day and position as additional explanatory parameters, and Tukey contrasts were calculated to compare treatments. The Wilcoxon paired test was used to compare fly landings on the blue and black portions of targets. Landing heights by each sex of *G. fuscipes fuscipes* on 1 m^2^ and 1.5 m^2^ targets were analysed using a Z-test of the log odds calculated using a multinomial logistic model [35], [36]. Kendall's rank correlation coefficient was used to test for a relationship between the length of target edge and fly landings on the upper, middle and lower blue and black sections of 1 m^2^ and 1.5 m^2^ targets in the 2011 Kenyan trial. Data for the two target sizes were analysed jointly.

## Results

### Best trapping device and blue material

When unbaited, the targets covered with adhesive film were the best devices for *G. f. fuscipes* in Kenya and Ethiopia. They regularly captured four to six times more flies than biconical traps, irrespective of season in Kenya (P<0.001, Table 2). The targets in the unbaited Sudanese trials were also the best performing devices. Catches at this site were low and differences were not significant (P>0.05, Table 2). Biconical and pyramidal traps caught similar numbers of *G. f. fuscipes* in the Sudan (P>0.05; Table 2). Similar patterns were recorded for *G. tachinoides* in Ethiopia: the target covered in adhesive film caught more than twice as many flies as the biconical trap and more than three times as many flies as the monoconical trap (P<0.05, Table 2). Catches in biconical and monoconical traps were similar (P>0.05, Table 2).

**Table 2:** Detransformed^1^ mean daily catches^2^ of *G. f. fuscipes* and *G. tachinoides* with unbaited and POCA-baited trapping devices made of different blue fabrics and on transparent adhesive film set on its own.

		*G. f. fuscipes*								*G. tachinoides*	
		Mean daily catch								Mean daily catch	
		Kenya				Sudan		Ethiopia		Ethiopia	
		wet season		dry season		dry season		wet season		wet season	
Device	Blue material	unbaited	POCA	unbaited	POCA	unbaited	POCA	unbaited	POCA	unbaited	POCA
Biconical	standard	13.7 ^a^	13.9 ^a^	7.9 ^a^	12.8 ^a^	3.9 ^a^	2.9 ^ab^	6.4 ^a^	10.3 ^a^	12.5 ^a^	9.7 ^a^
	turquoise	22.0 ^a^	13.7 ^a^	7.5 ^a^	9.4 ^a^	3.6 ^a^	2.7 ^ab^				
Pyramidal	standard					4.0 ^a^	3.1 ^ab^				
	turquoise					4.4 ^a^	2.6 ^a^				
Monoconical	standard									7.9 ^a^	6.9 ^a^
Target 1 m^2^	standard					5.4 ^a^	5.4 ^bc^	32.6 ^b^	50.4 ^b^	27.2 ^b^	23.1 ^b^
	turquoise					5.7 ^a^	7.5 ^c^				
	local							33.3 ^b^	40.2 ^b^		
Target 1.5 m^2^	standard	96.4 ^b^	77.4 ^b^	55.4 ^b^	49.9 ^b^						
	turquoise	92.2 ^b^	51.0 ^b^	49.8 ^b^	49.7 ^b^						
Film only 1 m^2^	-							4.1 ^c^	6.9 ^c^	2.8 ^c^	5.5 ^c^

1 Transformed means and their standard errors are presented in Supplementary Table S1.

2 Means followed by the same letter in the same column are not significantly different (Tukey post hoc test, P  =  0.05).

For both species, the same trapping device made from different blue fabrics performed equally well (P>0.05; Table 2), and sex ratios were similar with the use of different blue fabrics with black. Targets caught more females (up to 1.3×more) compared to the 3-d traps.

### Performance of POCA-baited trapping devices

The relative rankings of POCA-baited devices were very similar to those in the unbaited trials for both *G. f. fuscipes* and *G. tachinoides*, with targets outperforming traps at all locations, including the Sudan. Targets captured around four times as many *G. f. fuscipes* as the best traps in Ethiopia and Kenya (P≤0.001; Table 2) and 1.8–3.5 times as many as in the Sudan (P≤0.01, turquoise target only; Table 2). The POCA bait did not affect the relative performance of the biconical compared to the pyramidal trap in the Sudan, with similar catches in both traps.

For *G. tachinoides*, the performance of the POCA-baited devices was nearly identical to the unbaited trials; i.e. the target covered in adhesive film caught more than twice as many flies as the biconical trap and more than three times as many flies as the monoconical trap (P<0.05 in both cases, Table 2). As in the unbaited trials, although the biconical trap caught more tsetse than the monoconical trap, this difference was not significant (P>0.05, Table 2) and there was also no difference between the performance of the same trapping device made from different blue fabrics (P>0.05). Sex ratios were unaffected by the blue material used on different devices. Targets caught up to 25–30% more females than traps.

### Testing adhesive film alone

The adhesive film, when used on its own as a target, caught few flies relative to the equivalent-sized cloth target covered with adhesive film. It caught 12–14% of the mean daily catch recorded for *G. f. fuscipes* and 10–23% of the mean daily catch for *G. tachinoides* on cloth targets (Table 2, P≤0.001). The sticky surface area of the targets was 2 m^2^ compared to 1 m^2^ for the adhesive film set on its own.

### Best landing device and optimal target size

#### Best landing device

**Figure 4:**
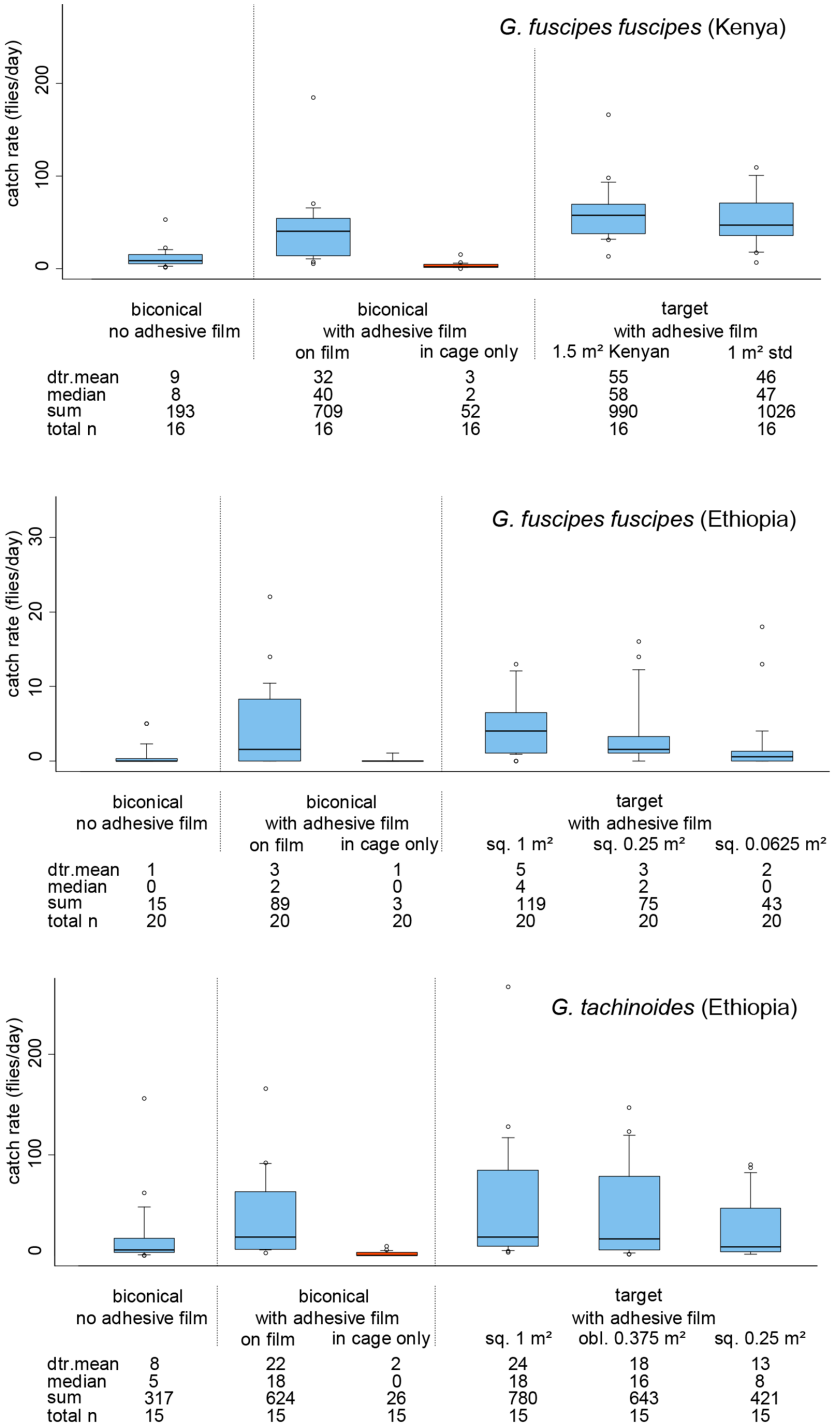
Daily catch rates of *G. f. fuscipes* and *G. tachinoides* by devices covered with adhesive film. Catch rates of traps are divided into fly catches on the cloth part and those trapped in the cage of the trap. The limits of the boxes indicate the twenty-fifth and seventy-fifth percentiles; the solid line in the box is the median; the capped bars indicate the tenth and the ninetieth percentiles, and data points outside these limits are plotted as circles: dtr. mean detransformed mean; obl. oblong; sq. square; std. standard.

At least a third more *G. f. fuscipes* landed on the targets than the biconical traps covered with adhesive film; differences were not significant (P>0.05, Figure 4). For *G. tachinoides*, numbers landing on the 1 m^2^ target and biconical traps were very similar, with only slightly more flies captured on the target (P>0.05; Figure 4). The relative number of females versus males landing was consistently greater on the 1 m^2^ targets (1.3–1.8 times more) compared with the traps for both species.

#### Optimal target size

The traditional Kenyan target, with a 50% greater surface area than the regular target only caught 17% more *G. f. fuscipes* per day in terms of detransformed means (P>0.05), with a very similar ratio of males to females on both targets. The arithmetic mean catch was actually higher on the regular target. The smaller targets deployed in Ethiopia caught fewer *G. f. fuscipes* and *G. tachinoides* than the regular target, but the reduced catch was only significant for the smallest targets tested (0.25 m×0.25 m and 0.5 m×0.5 m, respectively) in each experiment (P<0.01, Figure 4). When standardised to equal surface areas, the performance per unit area of the two smaller targets was better than the 1 m^2^ regular target by a factor of two to sevenfold for *G. f. fuscipes* and twofold for *G. tachinoides* (Table 3). However, the number of flies captured per metre edge of target remained almost constant across targets of varying size within experiments at different population densities for both species (Table 3).

**Table 3:** Catch indices (detransformed mean daily catch) per m^2^ and per m edge of different sized targets compared to a regular 1 m^2^ square target.

		*G. f. fuscipes*										*G. tachinoides*					
		Kenya					Ethiopia					Ethiopia					
Target		Mean daily catch	Catch index	P value	Flies per m^2^	Flies per m edge	Mean daily catch	Catch index	P value	Flies per m^2^	Flies per m edge	Mean daily catch	Catch index	P value	Flies per m^2^	Flies per m edge	Flies per m horiz. edge
1 m^2^	square	46	**1.0**		23.0	11.5	5	**1.0**		2.3	1.3	24	**1.0**		11.9	6.0	12
1.5 m^2^	oblong	55	**1.2**	**ns**	18.3	11.0	-										
0.375 m^2^	oblong	-					-					18	**0.8**	**ns**	24.7	7.2	12
0.25 m^2^	square	-					3	**0.6**	**ns**	6.4	1.5	13	**0.5**	**<0.01**	25.6	6.5	13
0.0625 m^2^	square	-					2	**0.4**	**<0.001**	15.2	2.0						

For *G. tachinoides*, the proportion of females captured declined slightly with decreasing target size, from 59% of the total catch on the 1 m^2^ target (P<0.05) to 48% on the smaller targets (P>0.05). In contrast, sex ratios remained constant on targets of different sizes for *G. f. fuscipes* (P>0.05), with the proportion of females ranging from 58–61% of the total catch.

### Preferential landing height

Landing was recorded at three heights for *G. f. fuscipes* on 1 m^2^ and 1.5 m^2^ targets in Kenya. Generally the fewest flies were caught in the middle on both the blue and black on both target sizes (Table 4). Statistically, more males were caught on the bottom third of the blue and black portions of the 1 m^2^ targets and females showed a preference for the top and bottom sections of the blue and black sections of the same targets (P<0.05 in all cases). Very similar landing height preferences were recorded on the blue and black sections of the larger rectangular 1.5 m^2^ targets, with preferences for the edges of the targets and avoidance of the middle by both sexes on both colours (Table 4). Analysis of fly landing data on the 1 m^2^ and 1.5 m^2^ targets in the 2011 Kenyan trial using Kendall's rank correlation coefficient indicates a strong correlation between the length of target section edges and fly landing responses (tau 0.21, z 5.01, P<0.001).

**Table 4:** Mean^1^ daily catch rate^2^ and percentage of total catch by sex at different landing heights on 1 m^2^ square (left) and 1.5 m^2^ rectangular (right) targets: *G. f. fuscipes*, Kenya (2011).

Colour	height	sex				Colour	height	sex			
1 m^2^		male	% of ♂catch	female	% of ♀catch	1.5 m^2^		male	% of ♂catch	female	% of ♀catch
Blue	top	**3.9 ^d^**	9.1	**7.1 ^b^**	12.0	Blue	top	**3.9 ^ab^**	15.1	**5.8 ^a^**	16.0
	middle	**4.2 ^cd^**	9.8	**2.6 ^c^**	4.4		middle	**2.1 ^b^**	8.1	**1.9 ^b^**	5.2
	bottom	**8.0 ^b^**	18.7	**5.0 ^b^**	8.4		bottom	**4.9 ^a^**	18.9	**4.2 ^a^**	11.6
Black	top	**6.7 ^bc^**	15.7	**16.8 ^a^**	28.3	Black	top	**4.5 ^a^**	17.4	**9.9 ^c^**	27.3
	middle	**8.1 ^b^**	19.0	**12.8 ^d^**	21.5		middle	**2.9 ^b^**	11.2	**5.7 ^a^**	15.7
	bottom	**11.8 ^a^**	27.7	**15.1 ^a^**	25.4		bottom	**7.6 ^c^**	29.3	**8.8 ^c^**	24.2
*N = 3,670 flies*						*N = 990 flies*					
*n = 27 targets*						*n = 12 targets*					

1 Standard errors of the means are presented in Supplementary Table S2

2 Means followed by the same letter (within same sex) are not significantly different (Tukey post hoc test, P  =  0.05)

### Colour preference

In the *G. f. fuscipes* trials there was a consistent preference for landings on the black portion of all but the smallest target for both sexes, and this preference was significant for females (Table 5, P<0.05, Wilcoxon test). There was no colour preference on the smallest target (0.25×0.25 m square). The sexes responded differently in the case of *G. tachinoides*, with males showing a preference for landing on the blue (with the exception of the smallest 0.5×0.5 m target), whilst females showed no clear preference for blue or black over the range of target sizes (Table 5).

**Table 5:** Medians^1^ of landing distribution of *G. f. fuscipes* and *G. tachinoides* between the blue and black portions of targets of different sizes.

	MALE daily catch		FEMALE daily catch		Blue/Black index^2^	
	Blue	Black	Blue	Black	Male	Female
***G. f. fuscipes***						
**Kenya (2011)**						
regular 1 m^2^	9	9.5	8.5	18	0.95	**0.47** **
Kenyan 1.5 m^2^	10	12	11.5	20	0.83	**0.58** **
regular 1 m^2^	10	22.5	7.5	34.5	0.44	**0.22** **
regular 1 m^2^	9.5	23.5	10.5	36.5	0.40	**0.29** **
regular 1 m^2^	18.5	22	14	36.5	0.84	**0.38** **
***G. tachinoides***						
**Ethiopia (2010)**						
regular 1 m^2^	14	7	2	1.5	**2.00** *	1.33
regular 1 m^2^	13.5	8	3.5	1.5	1.69	2.33
**Ethiopia (2012)**						
regular 1 m^2^	3	3	6	6	1.00	1.00
0.5×0.75 oblong	2	3	2	7	0.67	0.29
0.5×0.5 square	2	1	4	3	2.00	1.33

1 Median ranges are presented in Supplementary Table S3

2 P values following Wilcoxon test: * P < 0.05, ** P < 0.01

### Efficiency of biconical traps

Trapping efficiency in this study is defined as the flies caught in the cage as a proportion of the total number landing on/entering the trap. It was estimated by dividing the mean daily catch of the unaltered biconical trap by the mean daily catch of the matching traps with adhesive film on the cloth (flies caught on the adhesive film and in the cage; see Figure 4). This definition is conceptually different to studies working with e-nets, which is based on the interception of circling flies, due to the different nature of the measuring techniques. From these results, biconical trap efficiency was estimated at 25–26% for *G. f. fuscipes* and 33% for *G. tachinoides*.

## Discussion

### Comparison of trapping devices and fabrics

The results from Kenya, Sudan and Ethiopia suggest that targets made from appropriate black and blue fabrics (either phthalogen or turquoise) covered with adhesive film render them equal to or more efficient than traps at capturing *G. f. fuscipes* and *G. tachinoides*. This was clearly the case for *G. f. fuscipes* in both the wet and dry seasons in Kenya. In coordinated field trials in West Africa, we have already shown that 1 m^2^ black and blue targets covered with adhesive film capture 4–5 times more *G. tachinoides* than biconical traps [22]. Electric net results from interpretative experiments elsewhere in Africa [22], [26] imply that tsetse attraction to targets could be underestimated when adhesive film is used by up to 50% (for some riverine species). This may mean that targets are even better at attracting *G. f. fuscipes* and *G. tachinoides* to land than our East African data suggest. This is worth emphasising, as it is the landing response that underlies the principle of using insecticide-impregnated targets as control devices for tsetse. It is already well established for a range of tsetse species that only some of the flies attracted to the vicinity of traps or landing on their surfaces are eventually captured [18], [23], [26]. In the absence of a gold standard against which to assess the efficiency of electric nets and/or adhesive film in enumerating tsetse (e.g. by video observation), it is presently difficult to put confidence limits on the numbers of flies attracted to and/or landing on trapping devices as revealed by various techniques. To date these types of investigations have only been conducted for savannah and not riverine tsetse.

The use of insecticide-treated traps (pyramidal, monoconical and biconical) rather than targets remains the preferred control technique in many parts of East Africa for *G. f. fuscipes* and *G. tachinoides* [24], [37], [38]. Recent studies on *G. f. fuscipes* and on other riverine species elsewhere have now shown that targets and particularly small targets may be much more cost effective [18], [20], [23], [26]. Our results here support this general conclusion.

Aware of initial results from other studies, we therefore set out to better quantify how traps fare relative to targets at inducing a landing response for these two riverine species using different techniques. For this purpose another series of trials was conducted with both the targets and the cloth panels of the traps covered with adhesive film. This allowed us to explicitly count the number of flies landing on these devices, as we have done for other tsetse [22], [26], [25] (see below under performance of targets versus traps as landing devices below).

### Effects of POCA (on device performance)

The POCA chemical bait was used to test whether it increased trap efficiency as occurs for savannah tsetse [39]. Field trials with baited devices followed trials with unbaited devices at each location hence were not meant to compare unbaited and baited devices directly. However, bait had little or no obvious effect on improving trap entry (i.e. actual capture) relative to the excellent landing responses on targets for both *G. f. fuscipes* and *G. tachinoides*. Similarly, these baits did not improve relative rankings in terms of landing on targets versus traps. In fact the relative performance of devices remained remarkably constant between baited and unbaited trials conducted sequentially over a short time period (often less than a 5% difference for both species, with slight (10–15%) but non-significant increases in the proportion of males caught by the POCA-baited devices). This confirms earlier findings with chemical baited devices for *G. f. fuscipes* in Kenya [9] and *G. tachinoides* in West Africa [21], [22].

Similar studies in Ethiopia [40] with monoconical traps have come to similar conclusions. These authors showed that *G. tachinoides* was unresponsive to acetone or octenol alone. At best only modest increases in trap entry (up to double) could be achieved with a mixture of cow urine and octenol. This is in contrast to savannah species, such as *G. morsitans*, where captures up to ten times greater have been recorded with devices baited with natural and artificial attractants [41].

The relatively poor return on investment in increased catches for the cost/time involved in baiting devices for these riverine species would suggest that it could be more judicious to deploy additional targets in control campaigns rather than use and maintain chemical baits (with components such as phenols that are toxic to humans).

### Performance of regular targets versus biconical traps as landing devices

An unbaited 1 m^2^ adhesive film target caught relatively few flies relative to an equivalent regular target covered with adhesive film for both *G. f. fuscipes* and *G. tachinoides*, suggesting that the adhesive film alone is not inherently attractive to these riverine tsetse. Some attraction does occur under both baited and unbaited conditions (Table 2), but appears to be of similar magnitude to the inherent attraction that occurs when electric nets and associated apparatus are used for similar enumeration purposes [42]. Results with adhesive film alone for these riverine species are very similar to results for the savannah species *G. morsitans centralis* (10–13% of catch on sticky targets, unpublished data), but more than for *G. swynnertoni* and *G. pallidipes* (2% of catch on sticky targets [25]).

In the context of the potential efficiency of various devices, one third more *G. f. fuscipes* landed on the 1 m^2^ blue-black targets than on the outer surface (blue portion) of biconical traps in both Kenya and Ethiopia. The proportion was 20% higher on the target than on the outer surface (blue part) of the biconical trap for *G. tachinoides*. Since the biconical trap only had a cloth area of 0.7 m^2^ covered with adhesive film compared to 2 m^2^ on the target it would at first appear to be a rather efficient device at inducing landings for these two riverine species, with 1.7–2.5 times more flies per m^2^ landing on the traps. Indeed, Lindh *et al.* (2009) [23]found the biconical trap to fare as well as a 1 m^2^ black target for *G. f. fuscipes* in Kenya.

The low propensity of both species to enter the biconical trap without first landing on it also bodes well for its use as an insecticide-impregnated fly killing device. The proportion of *G. f. fuscipes* captured in the cage of biconical traps, i.e. those that entered the trap without first landing on its cloth, did not exceed 7% in either Kenya or Ethiopia. This proportion was 4% for *G. tachinoides* for the biconical trap, remarkably similar to the 7% recorded for the West African population of this species with the same trap part covered by adhesive film [22].

Interpretation of these results is complicated by the adhesive film which appears to affect the landing responses on the blue and black material. Our reflectance measurements from different cloths covered with adhesive film showed that the UV part of the light spectrum, which is known to adversely affect tsetse responses to objects, is absorbed. When used in field trials on visual control devices for *G. tachinoides* by Rayaisse *et al.* (2012) [22] and for *G. palpalis palpalis* by Kaba *et al.* (2014) [26] in West Africa, the adhesive film was found to reduce landings by half on 1 m^2^ blue-black targets. These insights were based on comparisons with electric net enumeration methods in the same experiments and were due to greatly reduced landings on the black portion of the target. Landings on the blue remained unchanged. Taking this phenomenon into account, fly numbers on adhesive-covered targets were almost certainly underestimated in this study by about half the actual numbers that would land on an unmodified target. In contrast, landings on the blue material of the biconical traps would be very similar between unmodified traps and those covered with adhesive film.

If we correct for the influence of the adhesive film on apparent landing rates, a direct comparison between the two devices shows that targets are still likely to be more efficient landing devices, with 2–2.5 times more *G. f. fuscipes* and 1.6 times more *G. tachinoides* landing on a target. Even allowing for the greater surface area of the targets, the numbers landing per m^2^ of cloth used to make the devices is nearly identical to those on the traps.

These results show that although insecticide-treated traps, which are still widely used against these species [24], are effective control devices, cheaper and easier to make targets are at least as good as and often better than more complicated and expensive traps. In light of this, further field experiments were designed to optimize target size and configuration as discussed below.

### Optimal target size, configuration and colour

#### *G. fuscipes fuscipes*

The 2011 field trial in Kenya showed that landing by *G. f. fuscipes* on the traditional 1.5 m^2^ Kenyan target was not different from a regular blue-black 1 m^2^ target. This confirms that there is no advantage in using this large local target, which has been used widely in Kenya since 2000 for savannah species. These results support work done by Esterhuizen *et al.* (2011) [18] with other riverine species. Reduced catches were recorded on smaller square targets in our trials in Ethiopia in 2012, but when counts are standardised for equal surface areas, these smaller devices proved to be more efficient per unit area at capturing *G. f. fuscipes* than the regular 1 m^2^ target by a factor of up to 6.5. Our results therefore support Lindh *et al.* (2009) [23] for this species on the shores of Lake Victoria in Kenya, and these authors also documented the cost effectiveness of using smaller targets in terms of the numbers of flies captured per unit area.

*G. f. fuscipes* has a clear preference for landing on the black, which would be even higher than our results indicate due to the influence of the adhesive film on landing on different colours (see previous section). However, a blue element is an essential part of any visual device to control this species, particularly in smaller targets as landings on the blue section can increase with decreasing target size as seen in Ethiopia, with a 25% increase in landing on the blue of the smallest targets (P<0.05, females only). Although the total catches in these trials were too small to draw firm conclusions on their own, they concur with Lindh et al. (2009) [23] and Esterhuizen *et al.* (2011) [18] that blue is a better attractant for small targets. Earlier findings by Green in 1989 [43] already highlighted the superior performance of bicoloured blue-black targets compared to all blue ones for another riverine species, *G. palpalis palpalis*.

On both colours, the Kenyan data indicated that fly landings were not evenly distributed by height but strongly correlated with the length of target section edges, with higher numbers caught around the longer upper and lower edges, i.e. with catches lowest in the middle section that comprise only 17% of the target border on 1 m^2^ targets. Exploiting edge effects in target design appears to be a fruitful area for more elaborate comparisons. Overall, males showed a landing bias for the bottom of the 1 m^2^ target (both colours) which is similar to the behaviour observed in other palpalis group species, notably *G. palpalis gambiensis*, where males land significantly lower than females [44]. This may be related to the size difference between the sexes, with the smaller males flying closer to the ground. The females showed an overall preference for the top and bottom of the blue section, with landing more generalised on the black. A preference for edges of objects as revealed by Brady and Shereni [45] and Doku and Brady [46] in *G. morsitans morsitans* may partially explain why smaller targets, with a higher edge/surface area ratio are more efficient at catching flies per unit area, provided that they are still large enough to attract flies and induce landing. These findings underline the importance of both the upper and lower edge of the target and its height for inducing landing. In certain size ranges, oblong (horizontal rectangular) targets, with their higher edge/surface area ratio and longer upper and lower edges may prove to be more efficient than equivalent sized square targets for *G. f. fuscipes*. The greater efficiency of rectangular targets in certain size ranges has been recorded for several tsetse species [25], is implied in the *G. tachinoides* data (below).

#### *G. tachinoides*

Smaller blue-black square or oblong targets covered with adhesive film caught over 2-fold more *G. tachinoides* per unit area in Ethiopia in 2012 than the regular 1 m^2^ target. Interestingly, catches per m^2^ were nearly identical between the 0.25 m^2^ square target and the 50% larger oblong. From other studies, we would have expected higher densities on the smaller square. As discussed above, this suggests that in these size ranges, a horizontal rectangle with a high edge/surface area ratio than the square and longer upper and lower edges is more efficient at inducing landing than a square. Indeed, for this particular data set, there is a very strong correlation between the numbers of *G. tachinoides* landing and the length of horizontal edge on the target.

Esterhuizen at al. (2011) [18]recorded a similar size-related trend for this species in Burkina Faso and, as in the study by Rayaisse *et al.* (2011) [20] working on the same population, catches on a 0.75×0.5 m oblong target made of blue cloth flanked by netting caught as many flies as a regular 1 m^2^ black-blue-black target. In the present study, the proportion of females captured declined with decreasing target size with a 20% drop in females recorded between the 1 m^2^ square target and the 0.25 m^2^ target. Esterhuizen *et al.* (2011) [18] also recorded a marked reduction in the number of females on very small targets in West Africa. Male *G. tachinoides* showed an overall preference for landing on the blue portion of targets whilst females' preference varied between devices. However, Rayaisse *et al.* (2012) [22] found that adhesive film reduces landing on the black and that both sexes showed a preference for the black on unmodified targets.

### Biconical trap monitoring efficiency

As anticipated from many studies on other tsetse species, the biconical trap caught only a moderate number of the *G. f. fuscipes* and *G. tachinoides* that landed on its attractive surfaces. Whereas the biconical trap is indeed capable of attracting substantial numbers of both *G. f. fuscipes* and *G. tachinoides* to land on it, the proportion of flies captured did not exceed 26% for *G. f. fuscipes* and 33% for *G. tachinoides*. One has to acknowledge that an accurate estimation of trapping efficiency is problematic due to the many unknowns about fly behaviour and counting efficiency near traps that could affect the outcome. Nevertheless, in field trials where trap efficiency was estimated using a different method, i.e. biconical traps and flanking electric nets, and definition (captured flies as a proportion of total number of tsetse attracted to the vicinity of the trap), Omolo *et al.* (2009) [9] recoded efficiency levels that did not exceed 20% for *G. f. fuscipes* females in Kenya. With our method, Rayaise *et al.* (2012) [22] recorded an identical efficiency level for the biconical trap in a West African population of *G. tachinoides* in Burkina Faso as that recorded here for the same trap in Ethiopia. As well as overall trap efficiency, the fact that fewer females enter traps than land on them (on average 30% less in this study) has important implications for the interpretation of trap catches when used for population monitoring.

### Concluding remarks

This study has confirmed that insecticide-treated biconical and monoconical traps are relatively effective control devices under a wide variety of conditions for *G. f. fuscipes* and *G. tachinoides*. Although not actually catching large numbers of flies, these types of traps induce a strong landing response, and hence achieve the desired endpoint of killing flies through the use of insecticide-impregnation, and are still favoured by many researchers as they allow tsetse population dynamics to be followed during a control programme. However, our study has shown that the deployment of smaller targets would be far more efficient, and economical. Such devices are also far more practical as they are less prone to wind damage and loss.

These conclusions concur with independent findings for *G. palpalis palpalis* [20], [26], [42] and on other riverine species [22], [23], are in agreement with various studies on *G. tachinoides* and *G. f. fuscipes* in their main range. This means that a standardized target of reduced size for use across Africa against these and other riverine tsetse species can be envisaged.

For small targets, oblong (horizontal rectangles) blue-black (not black only) devices appear to be the most efficient configuration to induce landing. The presence of blue (phthalogen or turquoise) is particularly important if very small targets are to be used. In theory, very small devices (0.0625 m^2^) would appear the most economical when comparing catches per m^2^ of material, but they can be rapidly hidden by re-growth of grasses and other ground vegetation, reducing visibility and landing rates [19]. A size of device which requires the application of herbicides adjacent to watercourses to keep it clear of vegetation is to be discouraged. The addition of flanking black netting to small targets (beyond the scope of this study) has been found to increase the proportion of *G. f. fuscipes* killed [23] but insecticide retention and life-span of the netting may be potential problems. The trial use of small targets with flanking netting in a control programme would provide answers to these concerns.

As small targets are less attractive to female *G. tachinoides*, we agree with Rayaisse et al (2011) that 0.5×0.75 m targets would be most suitable for this species, and on the balance of various practical considerations, also as a standardised target for both riverine species across their African range.

## Supporting Information

Figure S1 Reflectance spectra for phthalogen blue cloth used in the study, with and without adhesive film.
(TIF)

Figure S2 Reflectance spectra for turquoise blue cloth used in the study, with and without adhesive film.
(TIF)

Figure S3 Reflectance spectra for black cloth used in the study, with and without adhesive film.
(TIF)

Table S1 Detransformed mean daily catches (transformed means ± SEDs in brackets) of *G. f. fuscipes* and *G. tachinoides* with unbaited and POCA-baited devices.
(DOCX)

Table S2 Mean daily catch rate* (standard errors in brackets) at different landing heights on 1 m^2^ square (left) and 1.5 m^2^ rectangular (right) targets: *G. f. fuscipes*, Kenya (2011).
(DOCX)

Table S3 Medians (range in brackets) of landing distribution of *G. f. fuscipes* and *G. tachinoides* between the blue and black portions of targets of different sizes.
(DOCX)
