# Correction: Standardizing Visual Control Devices for Tsetse Flies: East African Species *Glossina fuscipes fuscipes* and *Glossina tachinoides*

**DOI:** 10.1371/journal.pntd.0003582

**Published:** 2015-03-19

**Authors:** The PLOS Neglected Tropical Diseases Staff

The figure legends for Fig. 2 and Fig. 3 are incorrect, they are currently switched. Please see the correct figure legends below.

**Fig 2:**
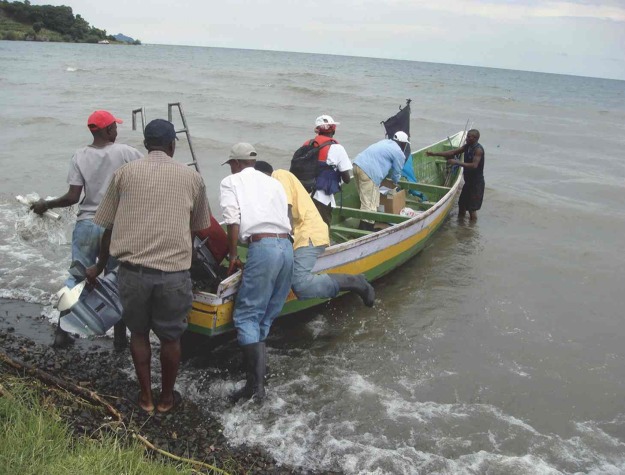
Transporting devices between trapping positions around Mfangano Island, Lake Victoria, Kenya.

**Fig 3:**
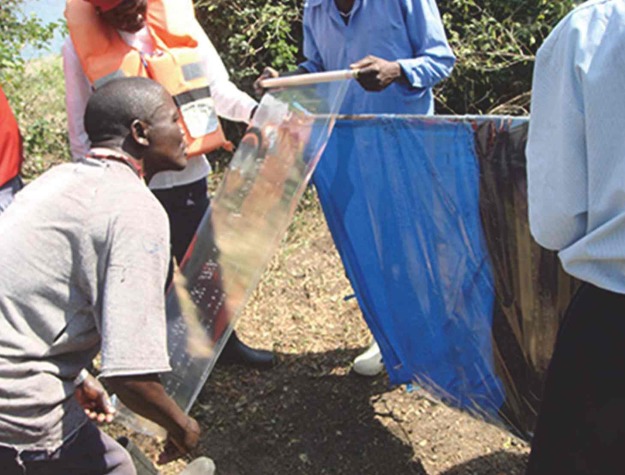
Placing adhesive film on bi-coloured target, Mfangano Island, Lake Victoria, Kenya.
