# Supplementary figures and images for: Standardizing Visual Control Devices for Tsetse Flies: East African Species Glossina fuscipes fuscipes and Glossina tachinoides
Source: PLoS Negl Trop Dis. 2014 Nov 20;8(11):e3334. doi: 10.1371/journal.pntd.0003334 (PMC4239017; doi:10.1371/journal.pntd.0003334)

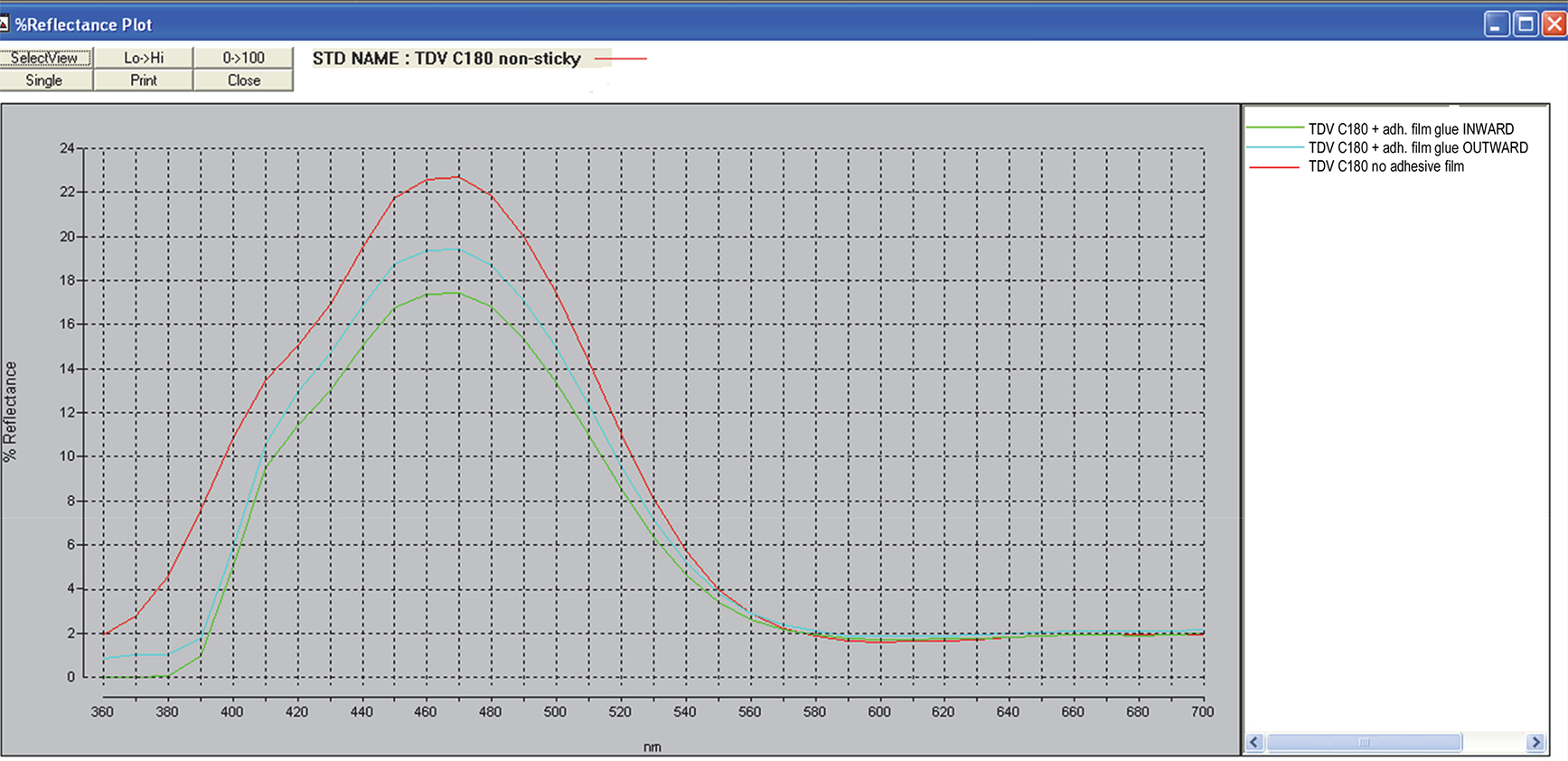

Supplement: Figure S1 — Reflectance spectra for phthalogen blue cloth used in the study, with and without adhesive film. (TIF) [file pntd.0003334.s001.tif]

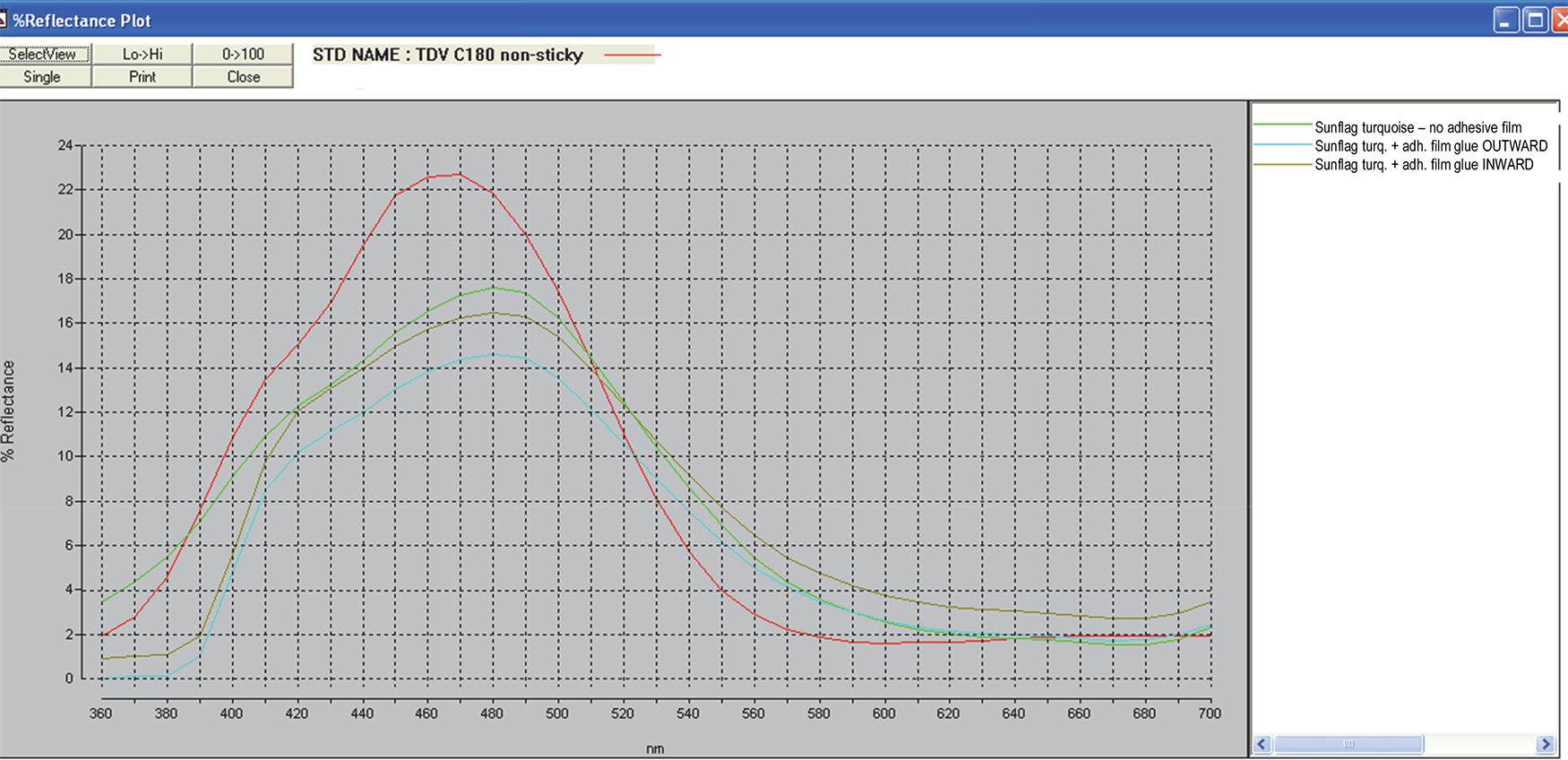

Supplement: Figure S2 — Reflectance spectra for turquoise blue cloth used in the study, with and without adhesive film. (TIF) [file pntd.0003334.s002.tif]

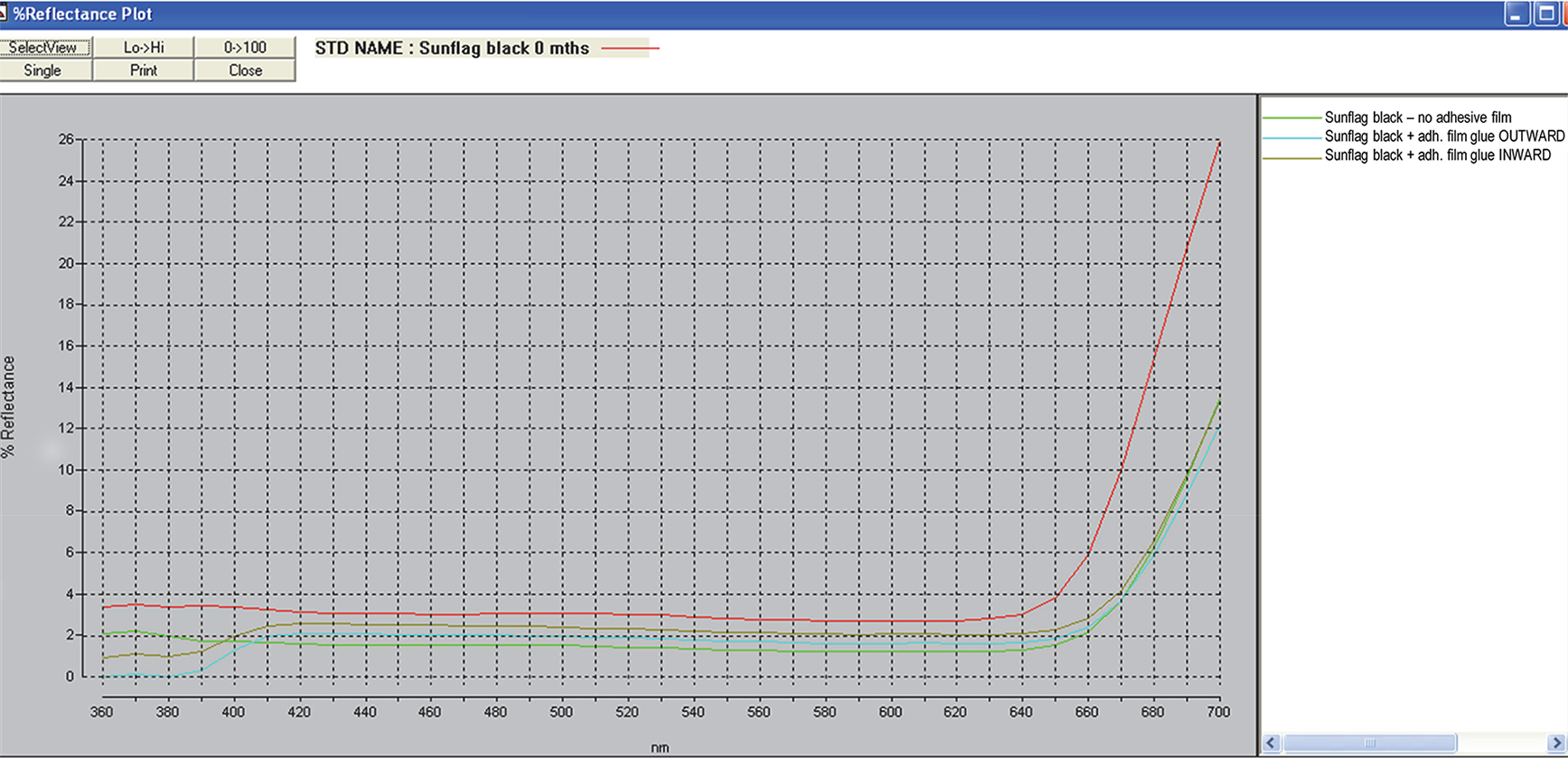

Supplement: Figure S3 — Reflectance spectra for black cloth used in the study, with and without adhesive film. (TIF) [file pntd.0003334.s003.tif]
