# Supplementary material for: Standardizing Visual Control Devices for Tsetse Flies: East African Species Glossina fuscipes fuscipes and Glossina tachinoides
Source: PLoS Negl Trop Dis. 2014 Nov 20;8(11):e3334. doi: 10.1371/journal.pntd.0003334 (PMC4239017; doi:10.1371/journal.pntd.0003334)
Supplement: Table S1 — Detransformed mean daily catches (transformed means ± SEDs in brackets) of G. f. fuscipes and G. tachinoides with unbaited and POCA-baited devices. (DOCX) [file pntd.0003334.s004.docx]

**Table S1. Detransformed mean daily catches (transformed means ± standard errors in brackets) of G. f. fuscipes and G. tachinoides with unbaited and POCA-baited devices**

|  |  | wet season |  | dry season |  |
| --- | --- | --- | --- | --- | --- |
| **Device** | **Blue material** | **unbaited** | **POCA** | **unbaited** | **POCA** |
| ***G. f. fuscipes*** |  |  |  |  |  |
| **Kenya** |  |  |  |  |  |
| Biconical | standard | 13.7 (2.61±0.206) | 13.9 (2.63±0.286) | 7.9 (2.06±0.379) | 12.8 (2.55±0.301) |
|  | turquoise | 22.0 (3.09±0.242) | 13.7 (2.61±0.235) | 7.5**^a^** (2.01±0.299) | 9.4 (2.24±0.313) |
| Target 1.5m^2^ | standard | 96.4 (4.57±0.193) | 77.4 (4.35±0.178) | 55.4 (4.01±0.190) | 49.9 (3.91±0.250) |
|  | turquoise | 92.2 (4.52±0.183) | 51.0 (3.93±0.205) | 49.8 (3.91±0.202) | 49.7 (3.91±0.257) |
| **Sudan** |  |  |  |  |  |
| Biconical | standard |  |  | 3.9 (1.37±0.174) | 2.9 (1.07±0.220) |
|  | turquoise |  |  | 3.6 (1.27±0.165) | 2.7 (0.99±0.188) |
| Pyramidal | standard |  |  | 4.0 (1.38±0.158) | 3.1 (1.15±0.210) |
|  | turquoise |  |  | 4.4 (1.48±0.211) | 2.6 (0.94±0.228) |
| Target 1 m^2^ | standard |  |  | 5.4 (1.68±0.132) | 5.4 (1.69±0.239) |
|  | turquoise |  |  | 5.7 **(**1.73±0.195) | 7.5 (2.02±0.193) |
| **Ethiopia** |  |  |  |  |  |
| Biconical | standard | 6.4 (1.86±0.087) | 10.3 (2.33±0.074) |  |  |
| Target 1 m^2^ | standard | 32.6 (3.48±0.073) | 50.4 (3.92±0.076) |  |  |
|  | local | 33.3 (3.50±0.100) | 40.2 (3.69±0.073) |  |  |
| Film only 1m^2^ | - | 4.1 (1.40±0.072) | 6.9 (1.92±0.108) |  |  |
| ***G. tachinoides*** | |  |  |  |  |
| **Ethiopia** |  |  |  |  |  |
| Biconical | standard | 12.5 (2.53±0.261) | 9.7 (2.28±0.358) |  |  |
| Monoconical | standard | 7.9 (2.06±0.272) | 6.9 (1.93±0.299) |  |  |
| Target 1 m^2^ | standard | 27.2 (3.30±0.168) | 23.1 (3.14±0.308) |  |  |
| Film only 1m^2^ | - | 2.8 (1.03±0.224) | 5.5 (1.70±0.164) |  |  |
