# Supplementary material for: Standardizing Visual Control Devices for Tsetse Flies: East African Species Glossina fuscipes fuscipes and Glossina tachinoides
Source: PLoS Negl Trop Dis. 2014 Nov 20;8(11):e3334. doi: 10.1371/journal.pntd.0003334 (PMC4239017; doi:10.1371/journal.pntd.0003334)
Supplement: Table S2 — Mean daily catch rate* (standard errors in brackets) at different landing heights on 1 m2 square (left) and 1.5 m2 rectangular (right) targets: G. f. fuscipes, Kenya (2011). (DOCX) [file pntd.0003334.s005.docx]

**Table S2**. Mean daily catch rate (standard errors in brackets) at different landing heights on 1 m^2^ square (left) and 1.5m² rectangular (right) targets: G. f. fuscipes, Kenya (2011).

| **Colour** | **height** | **sex** |  | **Colour** | **height** | **sex** |  |
| --- | --- | --- | --- | --- | --- | --- | --- |
| 1 m² |  | **male** | **female** | 1.5 m² |  | **male** | **female** |
| Blue | top | **3.9** (0.7) | **7.1** (1.2) | Blue | top | **3.9** (0.8) | **5.8** (1.2) |
|  | middle | **4.2** (0.7) | **2.6** (0.5) |  | middle | **2.1** (0.5) | **1.9** (0.5) |
|  | bottom | **8.0** (1.3) | **5.0** (0.9) |  | bottom | **4.9** (1.0) | **4.2** (0.9) |
| Black | top | **6.7** (1.1) | **16.8** (2.7) | Black | top | **4.5** (0.9) | **9.9** (1.9) |
|  | middle | **8.1** (1.4) | **12.8** (2.1) |  | middle | **2.9** (0.7) | **5.7** (1.1) |
|  | bottom | **11.8** (1.9) | **15.1** (2.5) |  | bottom | **7.6** (1.5) | **8.8** (1.7) |
|  |  |  |  |  |  |  |  |
| *N = 3,670 flies* | | | | *N = 990 flies* | | | |
| *n = 27 targets* | | | | *n = 12 targets* | | | |
