# Supplementary material for: Standardizing Visual Control Devices for Tsetse Flies: East African Species Glossina fuscipes fuscipes and Glossina tachinoides
Source: PLoS Negl Trop Dis. 2014 Nov 20;8(11):e3334. doi: 10.1371/journal.pntd.0003334 (PMC4239017; doi:10.1371/journal.pntd.0003334)
Supplement: Table S3 — Medians (range in brackets) of landing distribution of G. f. fuscipes and G. tachinoides between the blue and black portions of targets of different sizes. (DOCX) [file pntd.0003334.s006.docx]

**Table S3**. Medians (range in brackets) of landing distribution of G. f. fuscipes and G. tachinoides between the blue and black portions of targets of different sizes.

|  | **MALE daily catch** | | **FEMALE daily catch** | | **Blue/Black index#** | |
| --- | --- | --- | --- | --- | --- | --- |
|  | **Blue** | **Black** | **Blue** | **Black** | **Male** | **Female** |
| ***G. f. fuscipes*** |  |  |  |  |  |  |
| **Kenya (2011)** |  |  |  |  |  |  |
| regular 1 m² | **9** (1-38) | **9.5** (0-77) | **8.5** (1-39) | **18** (1-136) | 0.95 | **0.47**** |
| Kenyan 1.5 m² | **10** (2-31) | **12** (5-54) | **11.5** (2-24) | **20** (0-58) | 0.83 | **0.58**** |
| regular 1 m² | **10** (1-68) | **22.5** (1-108) | **7.5** (0-40) | **34.5** (7-133) | 0.44 | **0.22**** |
| regular 1m² | **9.5** (2-41) | **23.5** (0-53) | **10.5** (1-53) | **36.5** (10-105) | 0.40 | **0.29**** |
| regular 1m² | **18.5** (0-31) | **22** (1-69) | **14** (1-49) | **36.5** (4-82) | 0.84 | **0.38**** |
| ***G. tachinoides*** | | | | | | |
|  |  |  |  |  |  |  |
| **Ethiopia (2010)** |  |  |  |  |  |  |
| regular 1m² | **14** (6-45) | **7** (1-22) | **2** (0-11) | **1.5** (0-18) | **2.00*** | 1.33 |
| regular 1m² | **13.5** (0-29) | **8** (0-18) | **3.5** (0-5) | **1.5** (0-5) | 1.69 | 2.33 |
| **Ethiopia (2012)** |  |  |  |  |  |  |
| regular 1m² | **3** (0-46) | **3** (0-56) | **6** (0-57) | **6** (1-108) | 1.00 | 1.00 |
| 0.5 x 0.75 oblong | **2** (0-81) | **3** (0-38) | **2** (0-31) | **7** (0-50) | 0.67 | 0.29 |
| 0.5 x 0.5 square | **2** (0-37) | **1** (0-20) | **4** (0-24) | **3** (0-20) | 2.00 | 1.33 |

# P values following Wilcoxon test: * P < 0.05, ** P < 0.001, *** P < 0.001
